# 1277. Antimicrobial Resistance in Periprosthetic Joint Infection over a Decade

**DOI:** 10.1093/ofid/ofad500.1117

**Published:** 2023-11-27

**Authors:** Judith Alvarez Otero, Melissa J Karau, Kerryl E Greenwood-Quaintance, Jayawant Mandrekar, Matthew P Abdel, Robin Patel

**Affiliations:** Mayo Clinic, Rochester, Minnesota; Mayo Clinic, Rochester, Minnesota; Mayo Clinic, Rochester, Minnesota; Mayo Clinic, Rochester, Minnesota; Mayo Clinic, Rochester, Minnesota; Mayo Clinic, Rochester, Minnesota

## Abstract

**Background:**

Periprosthetic joint infection (PJI) is associated with significant morbidity. Management includes surgery and prolonged antibiotic treatment. Few studies have evaluated rates of acquired antimicrobial resistance in bacteria causing PJI. The aim of this study was to describe antimicrobial resistance profiles of bacteria causing PJI and to assess whether there was any apparent association between acquired resistance and clinical outcome.

**Methods:**

Patients at Mayo Clinic with first episodes of knee or hip PJI whose implants underwent sonication between January 2012 and December 2021 were studied. Sonicate fluid cultures from cement spacers or cultures yielding more than one microorganism were excluded. PJI was defined according to IDSA guidelines. Multidrug resistance was defined as non-susceptibility to at least one agent in three or more antimicrobial classes. Outcomes of PJI recurrence, need for further surgery with negative cultures or cultures yielding a new microorganism and amputation were assessed, within one year. Analysis was performed using SAS software version 9.4 (SAS Inc).

**Results:**

256 PJI episodes were evaluated, 55% from knees and 45% from hips. Median patient age was 66 years, 52% were men. Resistance profiles are shown in the table. The most frequent microorganisms isolated were *S. epidermidis* (n=82) and *S. aureus* (n=50), of which 68% and 30%, respectively, were methicillin resistant. Streptococci, Enterococci, Enterobacterales, and *P. aeruginosa* were found in 34,16, 8 and 3 cases; none were multidrug-resistant. No differences in PJI recurrence within one year were found in subjects with methicillin-resistant compared to methicillin-susceptible staphylococci (*S. aureus*, 50% *versus* 50%, respectively, p, 0.44; *S. epidermidis,* none), need for further surgery with negative cultures (*S. aureus*, 20% *versus* 80%, p, 0.72; *S. epidermidis,* 80% *versus* 20%, p, 0.47) or with cultures yielding a new microorganism (*S. aureus*, none; *S. epidermidis*, 67% *versus* 33%, p, 0.96), or amputation (none in any subject with staphylococcal PJI).

**Figure:**
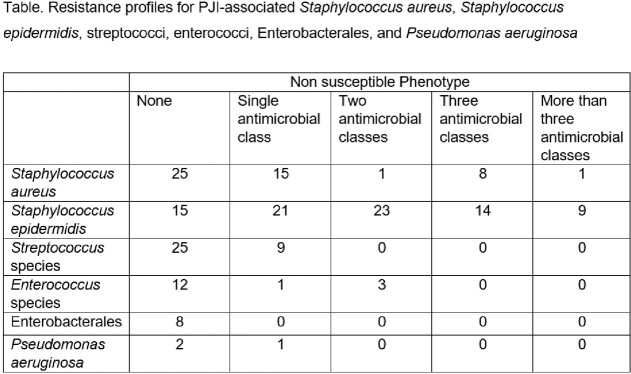

**Conclusion:**

Acquired antimicrobial resistance in PJI was most common in *S. epidermidis* with methicillin resistance found in 68% and multidrug resistance in 28%.

**Disclosures:**

**Matthew P Abdel, MD**, OsteoRemedies: Royalties|Springer: Publishing royalties|Stryker: Royalties reltated to hip and knee implants **Robin Patel, MD**, Abbott Laboratories: Advisor/Consultant|Adaptive Phage Therapeutics: Grant/Research Support|Adaptive Phage Therapeutics: Mayo Clinic has a royalty-bearing know-how agreement and equity in Adaptive Phage Therapeutics.|BIOFIRE: Grant/Research Support|CARB-X: Advisor/Consultant|ContraFect: Grant/Research Support|Day Zero Diagnostics: Advisor/Consultant|HealthTrackRx: Advisor/Consultant|Mammoth Biosciences: Advisor/Consultant|Netflix: Advisor/Consultant|Oxford Nanopore Technologies: Advisor/Consultant|PhAST: Advisor/Consultant|See details: Patent on Bordetella pertussis/parapertussis PCR issued, a patent on a device/method for sonication with royalties paid by Samsung to Mayo Clinic|See details: continued, patent on an anti-biofilm substance issued|TenNor Therapeutics Limited: Grant/Research Support|Torus Biosystems: Advisor/Consultant|Trellis Bioscience, Inc.: Advisor/Consultant

